# Tanshinone IIA-Loaded Nanoparticle and Neural Stem Cell Therapy Enhances Recovery in a Pig Ischemic Stroke Model

**DOI:** 10.1093/stcltm/szac062

**Published:** 2022-09-19

**Authors:** Erin E Kaiser, Elizabeth S Waters, Xueyuan Yang, Madison M Fagan, Kelly M Scheulin, Sydney E Sneed, Savannah R Cheek, Julie Heejin Jeon, Soo K Shin, Holly A Kinder, Anil Kumar, Simon R Platt, Kylee J Duberstein, Hea Jin Park, Jin Xie, Franklin D West

**Affiliations:** Regenerative Bioscience Center, Athens, GA, USA; Biomedical and Health Sciences Institute, Athens, GA, USA; Animal and Dairy Science Department, College of Agricultural and Environmental Sciences, Athens, GA, USA; Regenerative Bioscience Center, Athens, GA, USA; Biomedical and Health Sciences Institute, Athens, GA, USA; Animal and Dairy Science Department, College of Agricultural and Environmental Sciences, Athens, GA, USA; Environmental Health Science Department, College of Public Health, Athens, GA, USA; Chemistry Department, Franklin College of Arts and Sciences, Athens, GA, USA; Regenerative Bioscience Center, Athens, GA, USA; Biomedical and Health Sciences Institute, Athens, GA, USA; Animal and Dairy Science Department, College of Agricultural and Environmental Sciences, Athens, GA, USA; Regenerative Bioscience Center, Athens, GA, USA; Biomedical and Health Sciences Institute, Athens, GA, USA; Animal and Dairy Science Department, College of Agricultural and Environmental Sciences, Athens, GA, USA; Regenerative Bioscience Center, Athens, GA, USA; Animal and Dairy Science Department, College of Agricultural and Environmental Sciences, Athens, GA, USA; Regenerative Bioscience Center, Athens, GA, USA; Nutritional Sciences Department, College of Family and Consumer Sciences, Athens, GA, USA; Regenerative Bioscience Center, Athens, GA, USA; Animal and Dairy Science Department, College of Agricultural and Environmental Sciences, Athens, GA, USA; Small Animal Medicine and Surgery Department, College of Veterinary Medicine, Athens, GA, USA; Regenerative Bioscience Center, Athens, GA, USA; Biomedical and Health Sciences Institute, Athens, GA, USA; Animal and Dairy Science Department, College of Agricultural and Environmental Sciences, Athens, GA, USA; Chemistry Department, Franklin College of Arts and Sciences, Athens, GA, USA; Regenerative Bioscience Center, Athens, GA, USA; Interdisciplinary Toxicology Program, College of Pharmacy, University of Georgia, Athens, GA, USA; Regenerative Bioscience Center, Athens, GA, USA; Animal and Dairy Science Department, College of Agricultural and Environmental Sciences, Athens, GA, USA; Nutritional Sciences Department, College of Family and Consumer Sciences, Athens, GA, USA; Regenerative Bioscience Center, Athens, GA, USA; Chemistry Department, Franklin College of Arts and Sciences, Athens, GA, USA; Regenerative Bioscience Center, Athens, GA, USA; Biomedical and Health Sciences Institute, Athens, GA, USA; Animal and Dairy Science Department, College of Agricultural and Environmental Sciences, Athens, GA, USA; Small Animal Medicine and Surgery Department, College of Veterinary Medicine, Athens, GA, USA

**Keywords:** ischemia, stroke, neural stem cells, nanoparticle drug delivery system, multiparametric MRI, pigs

## Abstract

Induced pluripotent stem cell-derived neural stem cells (iNSCs) are a multimodal stroke therapeutic that possess neuroprotective, regenerative, and cell replacement capabilities post-ischemia. However, long-term engraftment and efficacy of iNSCs is limited by the cytotoxic microenvironment post-stroke. Tanshinone IIA (Tan IIA) is a therapeutic that demonstrates anti-inflammatory and antioxidative effects in rodent ischemic stroke models and stroke patients. Therefore, pretreatment with Tan IIA may create a microenvironment that is more conducive to the long-term survival of iNSCs. In this study, we evaluated the potential of Tan IIA drug-loaded nanoparticles (Tan IIA-NPs) to improve iNSC engraftment and efficacy, thus potentially leading to enhanced cellular, tissue, and functional recovery in a translational pig ischemic stroke model. Twenty-two pigs underwent middle cerebral artery occlusion (MCAO) and were randomly assigned to a PBS + PBS, PBS + iNSC, or Tan IIA-NP + iNSC treatment group. Magnetic resonance imaging (MRI), modified Rankin Scale neurological evaluation, and immunohistochemistry were performed over a 12-week study period. Immunohistochemistry indicated pretreatment with Tan IIA-NPs increased iNSC survivability. Furthermore, Tan IIA-NPs increased iNSC neuronal differentiation and decreased iNSC reactive astrocyte differentiation. Tan IIA-NP + iNSC treatment enhanced endogenous neuroprotective and regenerative activities by decreasing the intracerebral cellular immune response, preserving endogenous neurons, and increasing neuroblast formation. MRI assessments revealed Tan IIA-NP + iNSC treatment reduced lesion volumes and midline shift. Tissue preservation and recovery corresponded with significant improvements in neurological recovery. This study demonstrated pretreatment with Tan IIA-NPs increased iNSC engraftment, enhanced cellular and tissue recovery, and improved neurological function in a translational pig stroke model.

## Graphical Abstract

**Figure:**
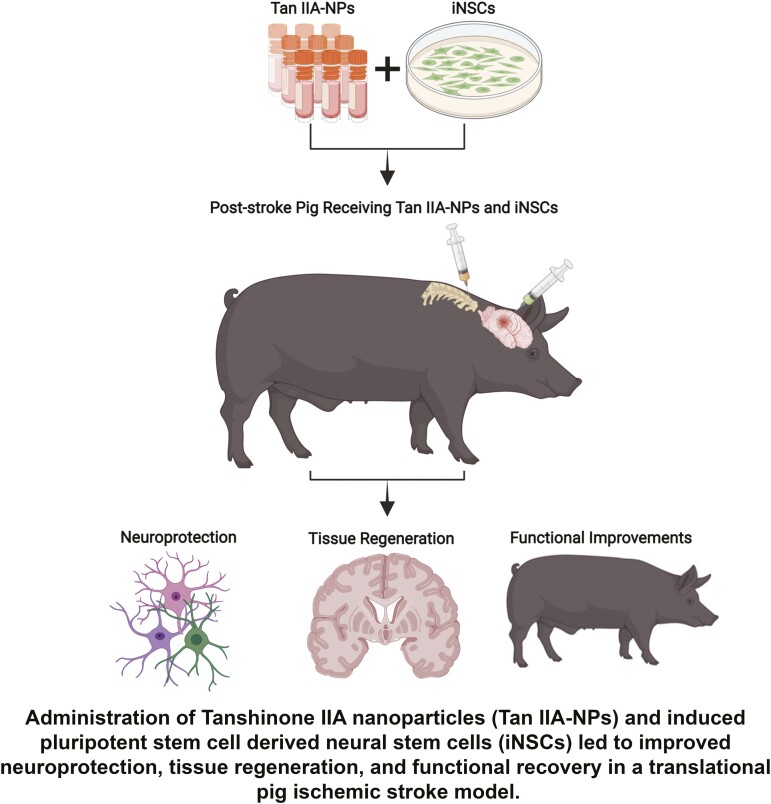

**Significance Statement**

This study presents the first experimental evidence pretreatment with antioxidative and anti-inflammatory Tanshinone IIA nanoparticles prior to induced pluripotent stem cell derived neural stem cell transplantation improves cell engraftment in pigs post-stroke. This novel combination therapy attenuated tissue degradation and stimulated neural recovery by increasing cell survival, neurogenesis, and neuroblast migration and decreasing immune cell activation and gliosis. Reductions in lesion volumes, midline shift, and white matter damage led to improved neurological recovery, thus suggesting that Tanshinone IIA nanoparticles could enhance the therapeutic effects of stem cell transplantation.

## Introduction

The lack of oxygen and nutrients to cerebral tissues following an ischemic stroke results in the destruction of approximately 1.9 million neurons, 14 billion synapses, and 12 km of myelinated fibers each minute a patient goes without treatment.^1^ Unfortunately, the only Food and Drug Administration (FDA) approved ischemic stroke therapies, tissue plasminogen activator (tPA) and endovascular thrombectomy, are available to a small subpopulation of patients due to acute treatment windows and associated risk factors.^2,3^ Furthermore, tPA and endovascular thrombectomy possess no direct regenerative capacity and cannot replace damaged cerebral tissue to enhance functional recovery. Recent studies in rodent and pig ischemic stroke models performed by our research team and others demonstrated induced pluripotent stem cell derived neural stem cells (iNSCs) are a multimodal therapeutic with neuroprotective, regenerative, and cell replacement properties that hold significant promise in promoting cellular, tissue, and functional recovery.^4-9^

iNSCs are an autologous regenerative cell replacement therapy that can be derived from a patient’s somatic cells (eg, skin fibroblasts), thus limiting the potential for rejection. In rodent ischemic stroke models, iNSCs differentiate into all 3 major neural cell types (eg, neurons, astrocytes, oligodendrocytes) and subtypes such as GABAergic and glutamatergic neurons that form neurites and synapses with endogenous neurons. Electrophysiological evaluation of these engrafted neurons demonstrated mature neuronal properties with the capability of sending and receiving synaptic input from host neurons.^10,11^ iNSCs in the pig ischemic stroke model have shown comparable differentiation potential and iNSCs in both rodent and pig models have demonstrated iNSCs produce a host of neuroprotective (eg, BDNF) and regenerative (eg, VEGF) factors that limit tissue damage and promote endogenous cerebral repair mechanisms.^5,6^ The combined therapeutic effects of iNSCs led to reduced lesion volumes and improved white matter integrity, brain metabolism, and cerebral perfusion in rodent and pig models.^4,5,12,13^ Collectively, these cellular and tissue level changes resulted in improvements in beam walking, grasping tasks, Morris water maze performance, postural reactions, and behavior. Despite these promising results, there is a substantial opportunity to improve the therapeutic efficacy of iNSCs as transplanted cells typically show low levels of engraftment.^5,12,14,15^ Increasing iNSC survival is likely to enhance long term engraftment and compound iNSC neuroprotective and regenerative signaling, thus increasing neural network performance and improving cognitive and motor recovery.

One of the major contributors to the limited long-term survivability and engraftment of iNSC derived neural cells is the highly cytotoxic stroke microenvironment which is characterized by elevated levels of inflammatory signaling and free radicals. Limiting the cytotoxicity in the stroked brain microenvironment will likely improve cellular engraftment. Tanshinone IIA (Tan IIA) is an antioxidative and anti-inflammatory agent that suppresses cytotoxicity in post-stroke brain tissue.^16,17^ Specifically, Tan IIA significantly increased the production of antioxidants (eg, superoxide dismutase) and reduced the production of pro-oxidants (eg, total nitric oxide synthase) in rodent ischemia/reperfusion models.^18-20^ Pretreatment with Tan IIA also protected against ischemia-induced neuronal death by increasing anti-inflammatory cytokines (eg, IL-4, 6, 8, 13) and decreasing inflammation through the PI3K/Akt/mTOR signaling pathway.^21,22^ Collectively, these antioxidative and anti-inflammatory effects resulted in attenuated cellular apoptosis, reduced lesion volumes, and improved neurological function in preclinical rodent studies as well as in our pig ischemic stroke model.^23-25^ Our research team recently demonstrated nanoparticle (NP)delivery of Tan IIA with FDA approved poly lactic-co-glycolic acid (PLGA) NPs (Tan IIA-NPs) may further improve the therapeutic potential of Tan IIA as ischemic stroke pigs treated with Tan IIA-NPs showed reduced lesion volumes, hemispheric swelling, midlineshift, hemorrhage, and white matter damage when compared with controls.^25^ By mitigating post-stroke inflammation and oxidative stress, pretreatment with Tan IIA-NPs may improve transplanted iNSC survivability and engraftment, thus increasing the neuroprotective and regenerative effects of iNSCs and promoting neurobehavioral and functional recovery.

To increase the translatability of a Tan IIA-NP and iNSC combination therapy based on the STAIRS and STEPS criteria, preclinical testing should be performed in a large animal model such as the pig ischemic stroke model.^26-28^ Compared with commonly used rodent models, pigs possess inherent anatomical similarities to humans that directly affect ischemic severity, penumbra evolution, and tissue recovery including gyrification, white and gray matter ratios, and collateral cerebral blood flow.^29^ Comparable cerebral volumes between pigs and humans (~10:1 ratio) allow for a more direct assessment of Tan IIA-NP and iNSC dosing.^30^ Despite the translational potential of non-human primate (NHP) models, there are several practical and scientific disadvantages including cost, housing facilities, veterinary care requirements, ethical challenges, the need for enucleation to access the middle and anterior cerebral arteries in some species, and species specific variance in cerebral vasculature and collaterals have limited NHP use in stroke research. These considerations are signifcantly less pronounced in pigs, thus making them an attractive alternative animal model.^31-33^ These attributes collectively support the use of a preclinical pig model to better predict treatment efficacy and consequent pathophysiological outcomes and to bridge the gap between rodent studies and human clinical trials.

The objective of this study was to evaluate the potential of Tan IIA-NPs to augment iNSC survival and engraftment and to facilitate iNSC cellular, tissue, and functional recovery post-stroke. For the first time, we present evidence Tan IIA-NPs enhanced iNSC derived neural cell survival and differentiation dynamics as well as improved endogenous neuroprotective and recovery mechanisms. These cellular effects not only led to decreased lesion volumes and midline shift, but also faster and higher levels of neurological recovery.

## Materials and Methods

### Study Design

Twenty-two pigs were randomly assigned to either PBS + PBS, PBS + iNSC, or iNSC + Tan IIA-NP experimental groups. The sample size for this study was determined by a power calculation based on our routine use of the pig middle cerebral artery occlusion (MCAO) model with magnetic resonance imaging (MRI)-based lesion volume being the primary endpoint. The power analysis was calculated using a 2-tailed analysis of variance (ANOVA) test, *α* = 0.05, and an 80% power of detection effect size of 1.19 and a standard deviation of 44.63. This was a blinded and randomized study in which 2 pigs were assigned to each surgical and MRI day. All endpoints and functional measurements were prospectively planned and underwent blinded analysis. Predefined exclusion criteria from all endpoints included instances of infection at the incision site, self-inflicted injuries that required euthanasia, inability to thermoregulate, uncontrolled seizure activity, and/or respiratory distress. No pigs were excluded from the study. No outliers were removed from the data.

## Results

### Tan IIA-NP Administration Improved iNSC Survivability

**Figure 1.**
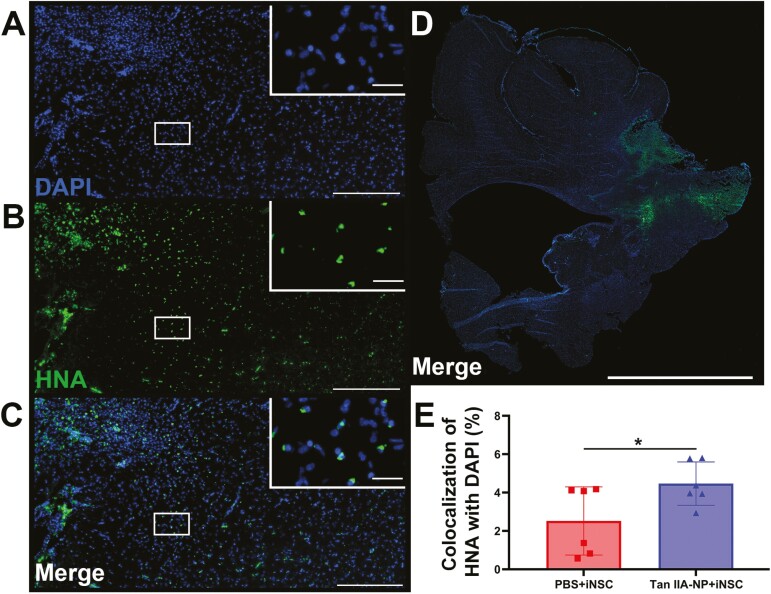
Tan IIA-NPs increased iNSC survival post-stroke. Twelve weeks post-transplantation, pigs were sacrificed and collected brain tissues underwent immunohistochemistry analysis to assess differences in iNSC survivability between PBS + iNSC and Tan IIA-NP + iNSC pigs. Human HNA + iNSCs colocalized with the cell nucleus marker DAPI at the lesion border (**A**-**C**; scale bar = 300 µm; white box scale bar = 10 µm). Quantitative analysis of ipsilateral hemisphere (**D**; scale bar = 10mm) revealed Tan IIA-NP treatment significantly improved survivability of transplanted iNSCs (4.47 ± 1.03%; **E**) relative to the PBS + iNSC control group (2.52 ± 1.62%). * indicates a significant (*P* < .05) difference between treatment groups. Data are expressed as mean ± SD. PBS + PBS data *n* = 6, PBS + iNSC data *n* = 6, and Tan IIA-NP + iNSC data *n* = 6.

To determine if Tan IIA-NPs improved human derived iNSC survivability, immunofluorescent staining was performed for the human cell marker HNA (Fig. 1B) and cell nuclear marker DAPI (Fig. 1A, merge 1C) 12 weeks post-transplantation. To control for cerebral size differences, HNA+/DAPI + iNSC-derived cell counts were normalized to the total number of DAPI + cells to create a %HNA + cells for each evaluated hemisphere. Whole hemisphere analysis (Fig. 1D) indicated Tan IIA-NP treated pigs possessed a significantly (*P* < .05) higher number of DAPI + cells that colocalized with HNA + cells (Fig. 1C) compared to PBS + iNSC treated pigs (4.47 ± 1.03 vs. 2.52 ± 1.62%; Fig. 1E). This suggests Tan IIA-NP administration increased iNSC engraftment post-stroke.

### Tan IIA-NPs Increased iNSC Neuronal Differentiation and Decreased iNSC Derived Reactive Astrocytes

**Figure 2.**
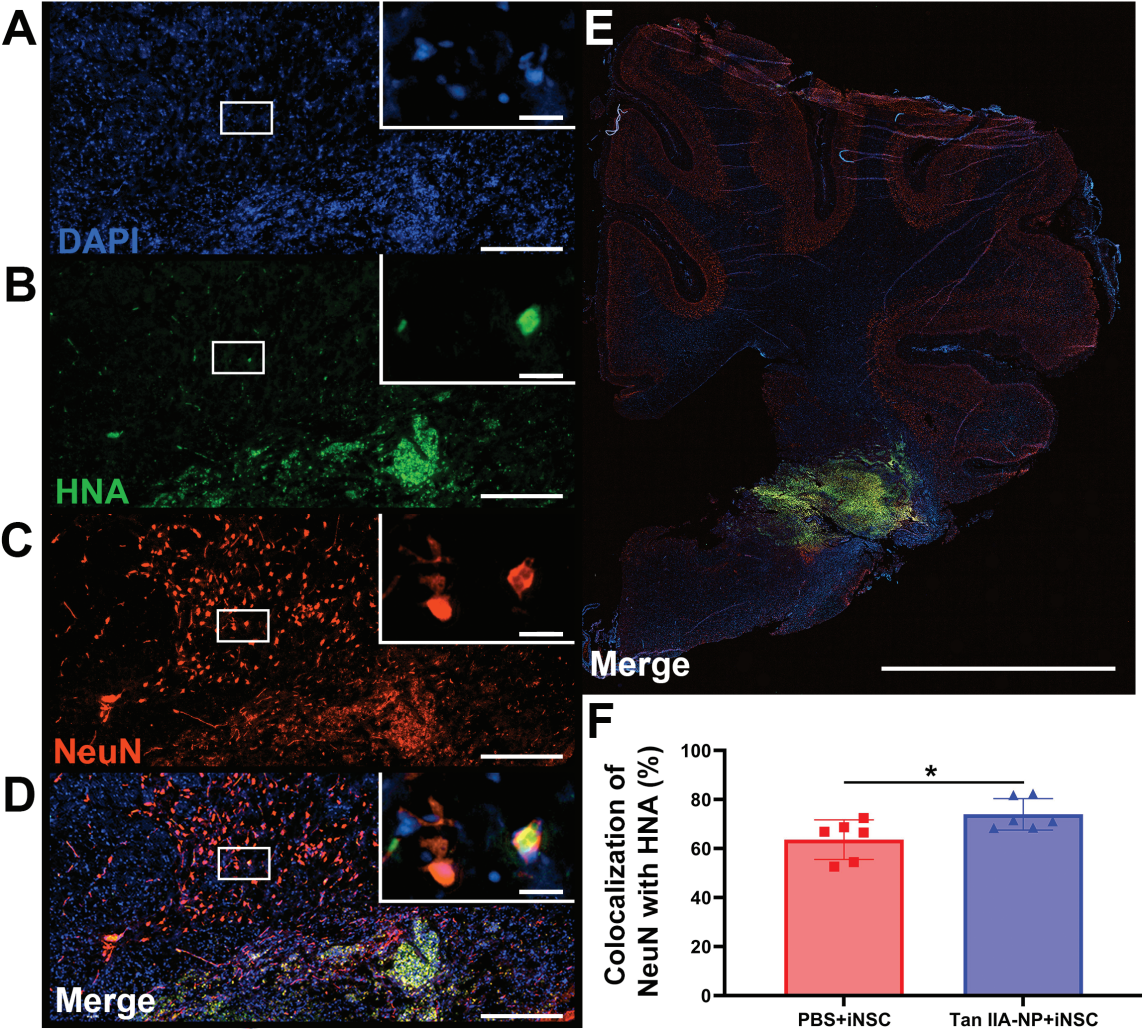
Tan IIA-NP treatment enhanced iNSC neuronal differentiation. Immunohistochemistry analysis showed human HNA + cells colocalized with the mature neuron protein NeuN at the lesion site 12 weeks post-transplantation (**A**-**D**; scale bar = 300 µm; white box; scale bar = 10 µm). Quantitative analysis of PBS + iNSC and Tan IIA-NP + iNSC ipsilateral hemisphere scans (**E**; scale bar = 10 mm) revealed Tan IIA-NPs increased the number of HNA+/NeuN + cells at 12 weeks post-transplantation (73.96 ± 5.86% vs. 63.61 ± 7.39%, respectively; **F**). Data are expressed as mean ± SD. * indicates a significant (*P* < .05) difference between treatment groups. PBS + PBS data *n* = 6, PBS + iNSC data *n* = 6, and Tan IIA-NP + iNSC data *n* = 6.

Additional immunofluorescence staining was performed for DAPI (Fig. 2A), HNA (Fig. 2B), and the neuronal marker NeuN (Fig. 2C). Again, the number of HNA+/DAPI+/NeuN + cells (Fig. 2D) were normalized to the number of DAPI + cells in each whole hemisphere slice (Fig. 2E) to account for differences in cerebral size. Quantification revealed Tan IIA-NP + iNSC treated pigs possessed significantly (*P* < .05) greater numbers of NeuN + cells that co-localized with DAPI+/HNA + than PBS + iNSC treated pigs (73.96 ± 5.86 vs. 63.61 ± 7.39%; Fig. 2F). These results suggest Tan IIA-NPs promote neuronal differentiation of transplanted iNSCs leading to replacement of damaged tissue post-stroke.

**Figure 3.**
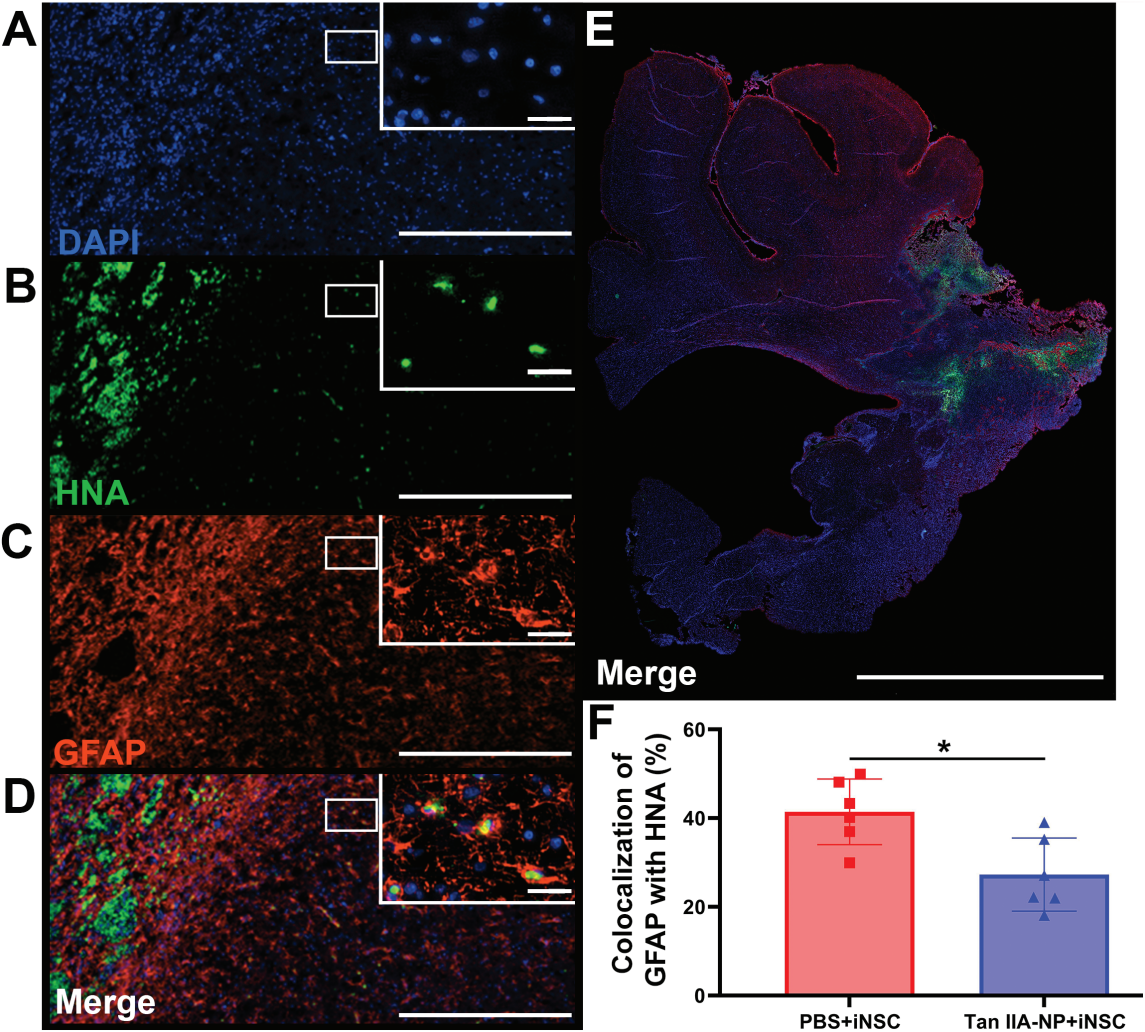
Tan IIA-NP treatment decreased iNSC differentiation into reactive astrocytes. HNA + cells colocalized with the reactive astrocyte protein GFAP and DAPI (**A**-**D**; scale bar = 300 µm; white box; scale bar = 10 µm). Quantitative analysis of PBS + iNSC and Tan IIA-NP + iNSC ipsilateral hemisphere (**E**; scale bar = 10mm) revealed Tan IIA-NP administration decreased the number of HNA+/GFAP + cells at 12 weeks post-transplantation (27.26 ± 7.53% vs. 41.41 ± 6.75%, respectively; **F**). Data are expressed as mean ± SD. PBS + PBS data *n* = 6, PBS + iNSC data *n* = 6, and Tan IIA-NP + iNSC data *n* = 6.

Co-localization of DAPI (Fig. 3A), HNA (Fig. 3B), and the reactive astrocyte marker GFAP (Fig. 3C) was also quantified in the ipsilateral hemisphere (Fig. 3E). Tan IIA-NP + iNSC treated pigs possessed significantly (*P* < .05) lower numbers of HNA+/DAPI+/GFAP + cells (Fig. 3D) compared to PBS + iNSC treated pigs (27.26 ± 7.53 vs. 41.41 ± 6.75%; Fig. 3F). These results suggest Tan IIA-NP administration may modulate the inflammatory ischemic stroke microenvironment, thus decreasing reactive astrocyte differentiation of transplanted iNSCs.

Immunofluorescent staining was also performed using the common oligodendrocyte marker Olig2. However, Olig2 staining could not be reliably quantified.

### Tan IIA-NP + iNSC Treatment Led to Neuronal Protection, Reduced Iba-1 + Immune Cell Activation, and Neuroblast Migration

**Figure 4.**
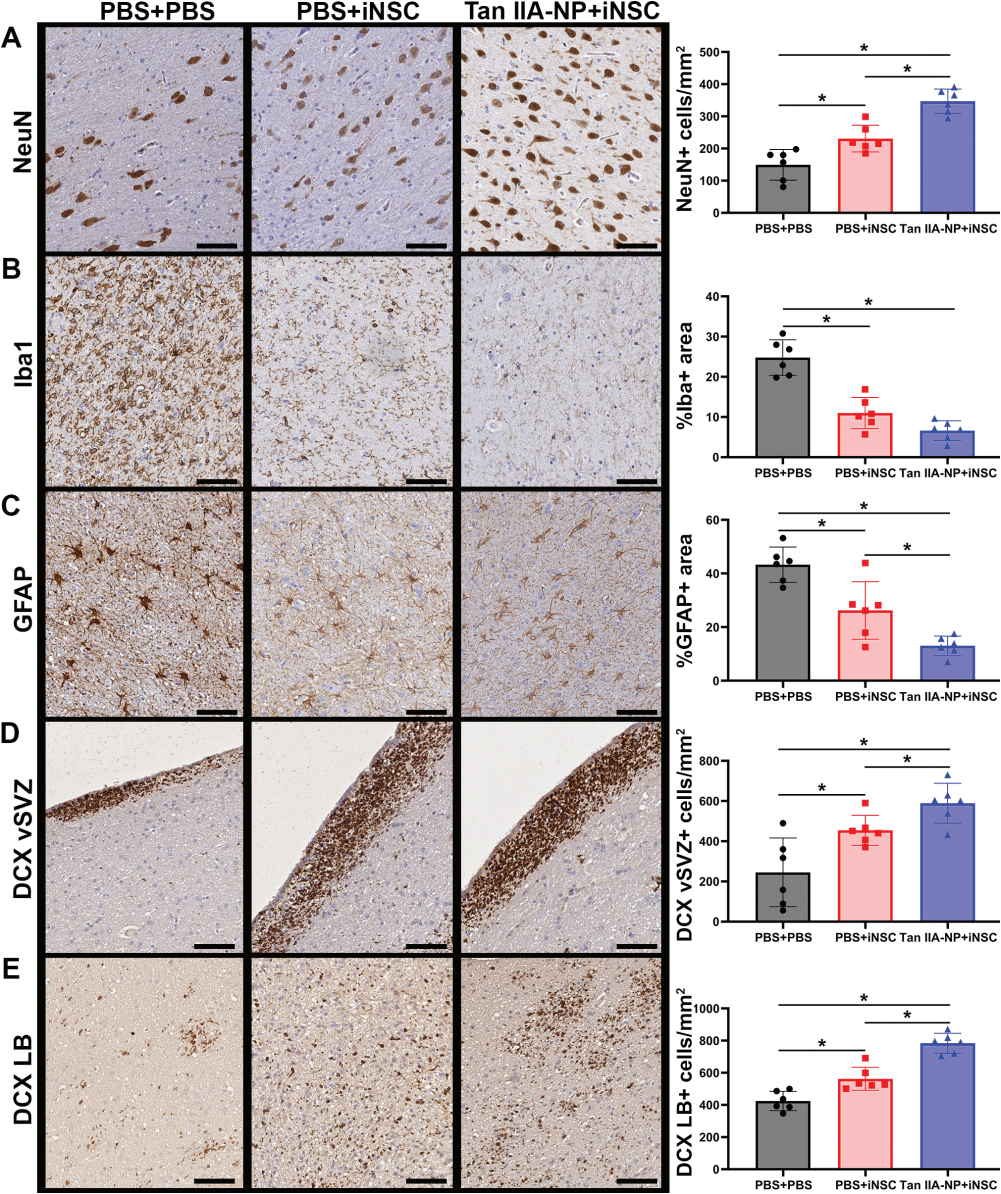
Tan IIA-NPs enhanced iNSC induced neuroprotective and regenerative activity. Tan IIA-NP + iNSC pigs showed an increased number of NeuN + neurons at 12 weeks post-transplantation relative to PBS + iNSC and PBS + PBS pigs (346.96 ± 34.45 cells/mm^2^ vs. 230.69 ± 37.81 cells/mm^2^ vs. 149.29 ± 43.57 cells/mm^2^, respectively; **A**). Tan IIA-NP + iNSC pigs also showed decreased numbers of Iba1 + immune cells relative to PBS + iNSC and PBS + PBS pigs (6.61 ± 2.21% vs. 10.98 ± 3.54% vs. 24.78 ± 4.05%, respectively; **B**). Similarly, Tan IIA-NP + iNSC pigs also showed fewer GFAP + reactive astrocytes relative to PBS + iNSC and PBS + PBS pigs (13.00 ± 3.36% vs. 26.20 ± 9.81% vs. 43.28 ± 6.02%, respectively; **C**). Tan IIA-NP + iNSC pigs demonstrated increased numbers of DCX + neuroblasts in the ventricular lining of the subventricular zone (vSVZ) relative to PBS + iNSC and PBS + PBS pigs (589.14 ± 90.49 cells/mm^2^ vs. 454.33 ± 68.36 cells/mm^2^ vs. 245.04 ± 156.11 cells/mm^2^, respectively; **D**) and at the lesion border (LB; 783.60 ± 56.69 cells/mm^2^ vs. 562.41 ± 65.01 cells/mm^2^ vs. 424.77 ± 54.70 cells/mm^2^, respectively; **E**). Scale bars 500 µm. Data are expressed as mean ± SD. PBS + PBS data *n* = 6, PBS + iNSC data *n* = 6, and Tan IIA-NP + iNSC data *n* = 6.

Next, the effects of Tan IIA-NPs and iNSCs on endogenous neuronal survival and immune response was evaluated 12 weeks post-transplantation. Tan IIA-NP + iNSC treated pigs showed a significant (*P* < .05) increase in the number of NeuN + neurons relative to PBS + iNSC and PBS + PBS treated pigs (346.96 ± 34.45 vs. 230.69 ± 37.81 vs. 149.29 ± 43.57cells/mm^2^; Fig. 4A). Tan IIA-NP + iNSC-treated pigs also showed a significant (*P* < .05) decrease in the number of Iba1 + immune cells (microglia, macrophages, and other infiltrating immune cells) relative to PBS + iNSC and PBS + PBS treated pigs (6.61 ± 2.21 vs. 10.98 ± 3.54 vs. 24.78 ± 4.05%; Fig. 4B). Similarly, Tan IIA-NP + iNSC-treated pigs also showed significantly (*P* < .05) fewer GFAP + reactive astrocytes relative to PBS + iNSC and PBS + PBS treated pigs (13.00 ± 3.36 vs. 26.20 ± 9.81 vs. 43.28 ± 6.02%; Fig. 4C). Post-stroke endogenous neural stem cells located in the subventricular zone (SVZ) have the ability to differentiate into immature neuronal cells, or neuroblasts, which migrate toward the site of injury to replace damaged and dead cells and release neuroprotective neurotrophic factors. Tan IIA-NP + iNSC treated pigs showed a significant (*P* < .05) increase in DCX + neuroblasts in the ventricular lining of the subventricular zone (vSVZ) relative to PBS + iNSC and PBS + PBS treated pigs (589.14 ± 90.49 vs. 454.33 ± 68.36 vs. 245.04 ± 156.11 cells/mm^2^; Fig. 4D) and at the lesion border (LB; 783.60 ± 56.69 vs. 562.41 ± 65.01 vs. 424.77 ± 54.70 cells/mm^2^; Fig. 4E). The PBS + iNSC group also showed a significant increase in NeuN + neuron survival and DCX + neuroblast formation at the vSVZ and LB and a significant decrease in GFAP + reactive astrocytes relative to the PBS + PBS treatment group. However, the Tan IIA-NP + iNSC group showed significant improvement in each of these metrics relative to the PBS + iNSC group.

### Tan IIA-NP + iNSC Treatment Decreased Lesion Volume and Consequential Midline Shift

**Figure 5.**
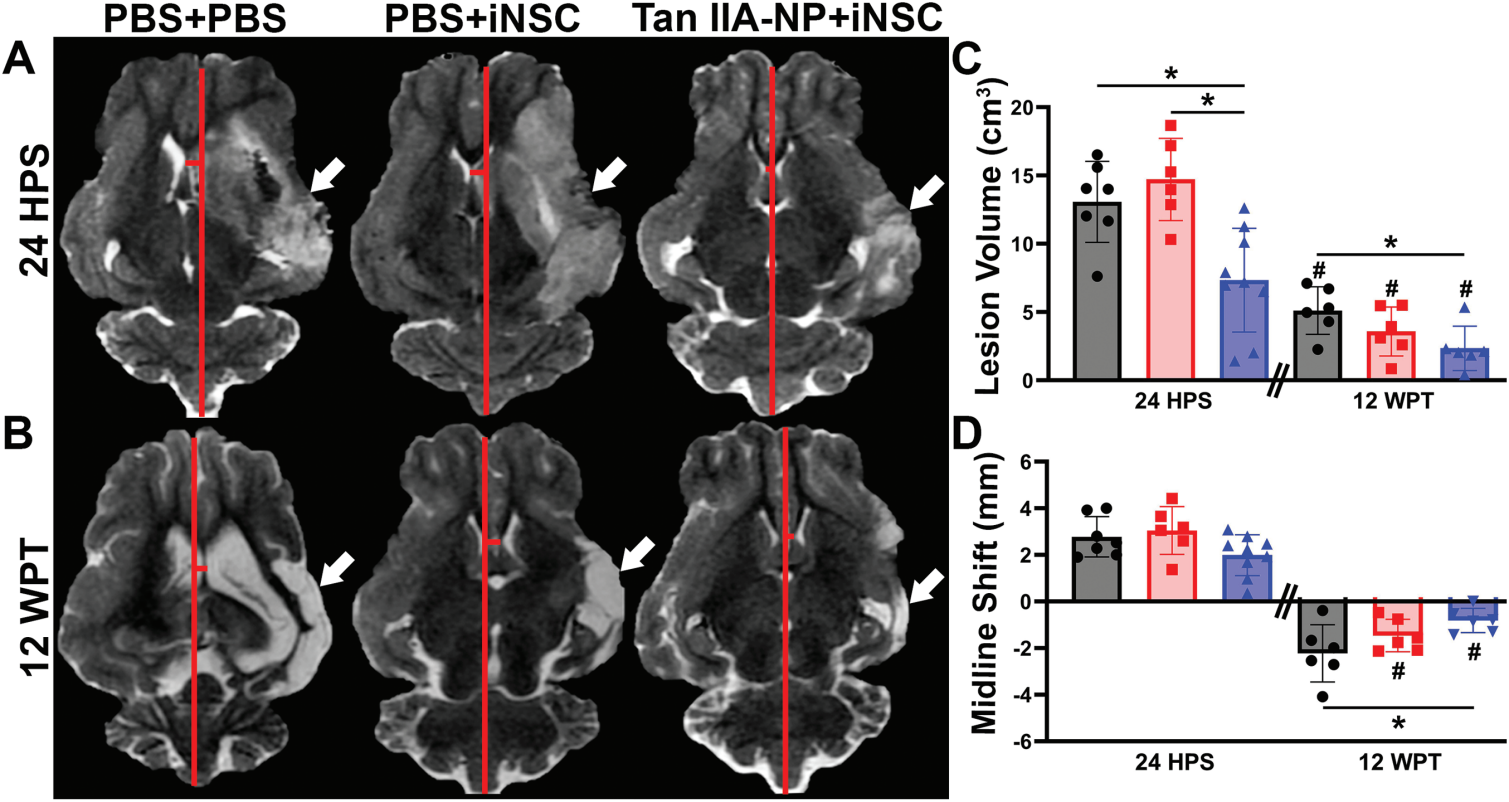
Tan IIA-NP + iNSC treatment decreased lesion volume and midline shift. Tan IIA-NP + iNSC pigs exhibited a significant decrease in hyperintense lesion volumes (white arrows) when compared with PBS + iNSC and PBS + PBS pigs at 24 hours post-stroke (24 HPS; 7.34 ± 1.27 cm^3^ vs. 14.72 ± 1.23 cm^3^ vs. 13.07 ± 1.12 cm^3^, respectively; **A**, **C**) and compared with PBS + PBS control pigs at 12 weeks post-transplantation (12 WPT; 2.35 ± 1.48 cm^3^ vs. 5.11 ± 1.59 cm^3^, respectively; **B**, C). All treatment groups possessed significantly reduced lesion volumes at 12 WPT when compared with individual 24 HPS values (5.11 ± 1.59 cm^3^ vs. 13.07 ± 2.75 cm^3^; 3.58 ± 1.64 cm^3^ vs. 14.72 ± 2.75 cm^3^; 2.35 ± 1.48 cm^3^ vs. 7.34 ± 3.58 cm^3^, respectively; C). Tan IIA-NP + iNSC pigs demonstrated a significantly less pronounced midline shift (red lines) 12 WPT compared with PBS + PBS control pigs (−0.81 ± 0.48 mm vs. −2.23 ± 1.12 mm, respectively; B, **D**) while Tan IIA-NP + iNSC and PBS + iNSC treated pigs also exhibited significant changes between time points (−0.81 ± 0.48 vs. 1.99 ± 0.82 mm; −1.46 ± 0.63 vs. 3.05 ± 0.93 mm, respectively; D). Data are expressed as mean ± SD. * indicates a significant (*P* < .05) difference between experimental groups. # indicates a significant (*P* < .05) difference between time points within an experimental group. At 24 HPS PBS + PBS data *n* = 7, PBS + iNSC data *n* = 6, and Tan IIA-NP + iNSC data *n* = 9. At 12 WPT PBS + PBS data *n* = 6, PBS + iNSC data *n* = 6, and Tan IIA-NP + iNSC data *n* = 6.

T2W sequences revealed significantly (*P* < .05) reduced hyperintense lesion volumes (white arrows; Fig. 5A) in Tan IIA-NP + iNSC-treated pigs when compared with PBS + iNSC and PBS + PBS control pigs 24 hours post-stroke (7.34 ± 3.58 vs. 14.72 ± 2.75 and 13.07 ± 2.75cm^3^; Fig. 5C). As iNSCs were not transplanted until 5 days post-stroke, this suggests that Tan IIA-NPs have an acute therapeutic effect. At 12 weeks post-transplantation, Tan IIA-NP + iNSC treated pigs exhibited significantly (*P* < .05) reduced lesion volumes compared with PBS + PBS control pigs (2.35 ± 1.48 vs. 5.11 ± 1.59 cm^3^; Fig. 5B) but not PBS + iNSC treated pigs, thus suggesting iNSCs alone also possess notable therapeutic effects. All experimental groups demonstrated significantly (*P* < .05) reduced lesion volumes at 12 weeks post-transplantation compared to 24 hours post-stroke lesion volumes (5.11 ± 1.59 vs. 13.07 ± 2.75; 3.58 ± 1.64 vs. 14.72 ± 2.75; 2.35 ± 1.48 vs. 7.34 ± 3.58 cm^3^; Fig. 5C).

At 24 hours post-stroke, all experimental groups exhibited ipsilateral hemispheric swelling, contralateral hemispheric compression, and thus a positive shift in midline (red lines). Although Tan IIA-NPs had no significant effect on midline shift 24 hours post-stroke, at 12 weeks post-transplantation Tan IIA-NP + iNSC treated pigs demonstrated a significant (*P* < .05) reduction in midline shift relative to PBS + PBS control pigs (−0.81 ± 0.48 vs. −2.23 ± 1.12 mm; Fig. 5D). This suggests reduced tissue atrophy in the ipsilateral hemisphere of Tan IIA-NP + iNSC treated pigs (Fig. 5B).

Twelve weeks post-transplantation, fractional anisotropy (FA) maps revealed Tan IIA-NP + iNSC and PBS + iNSC pigs exhibited a lower percent decrease in FA values compared with PBS + PBS control pigs (Supplementary Fig. S2). This suggests iNSCs may help mitigate white matter degradation following ischemic injury. At 24 hours post-stroke, all animals exhibited intracerebral hemorrhage (Supplementary Fig. S3). However, Tan IIA-NP + iNSC pigs demonstrated a significant (*P* < .05) reduction in intracerebral hemorrhage volumes and percent change in diffusivity when compared to PBS + iNSC and PBS + PBS control pigs.

### Tan IIA-NP + iNSC Treatment Improved Neurological Outcomes

**Figure 6.**
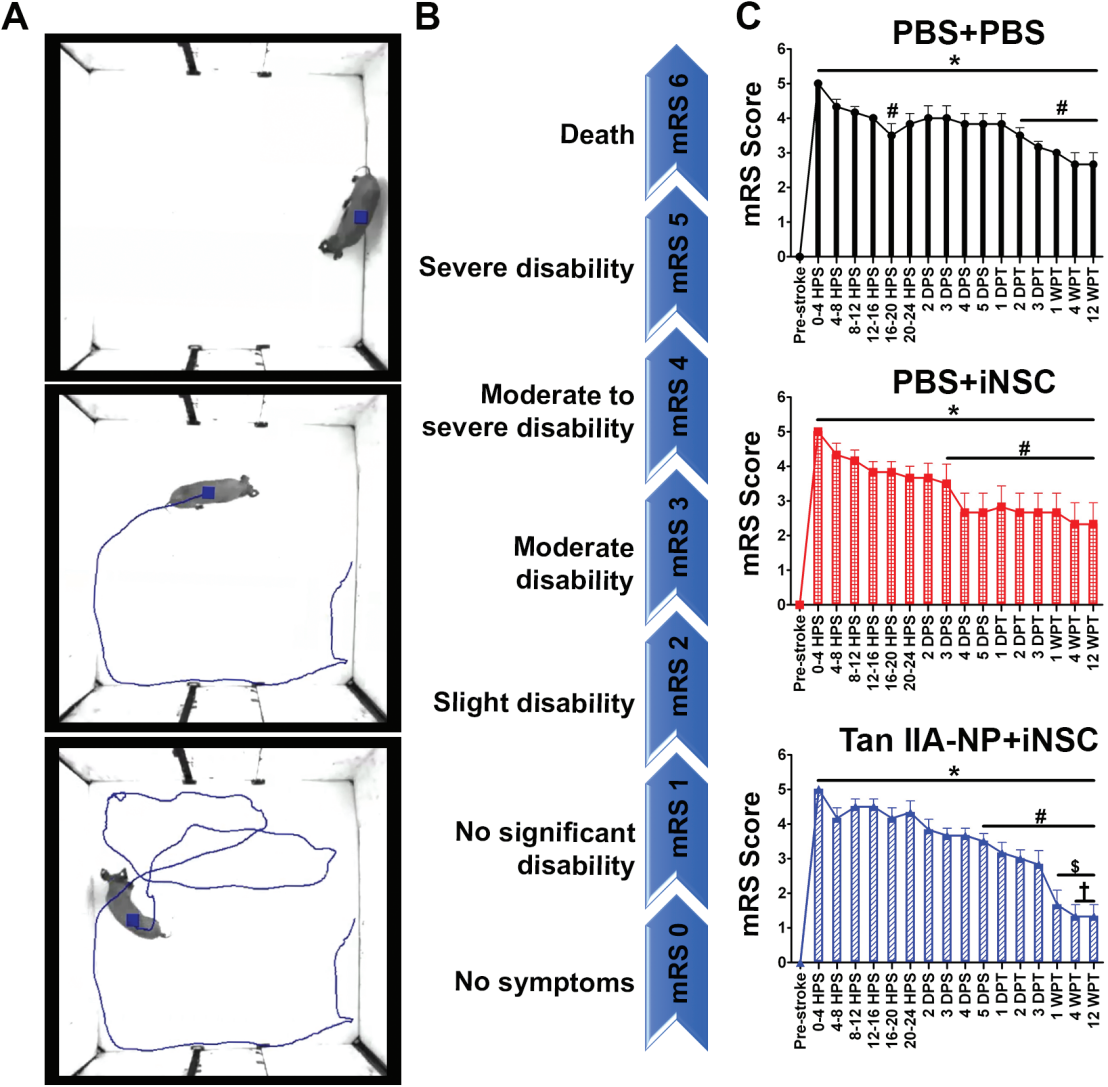
Tan IIA-NP + iNSC treatment improved post-stroke neurological recovery. Post-stroke assessments showed animals demonstrated acute severe disability (top image; **A**) and improved recovery over time (bottom image). Post-stroke symptoms were assigned a modified Rankin Scale (mRS) score ranging from 0 (no symptoms) to 6 (death; **B**). mRS scores revealed Tan IIA-NP + iNSC pigs demonstrated decreased mRS scores at 1 week post-transplantation (WPT; **C**) relative to 5 days post-stroke (DPS) and 1 day post-transplantation (DPT) and at 4 and 12 WPT relative to 2 and 3 DPT. Comparatively, PBS + iNSC and PBS + PBS pigs never demonstrated this level of neurological recovery. At 1, 4, and 12 WPT Tan IIA-NP + iNSC pigs possessed significantly reduced scores compared to PBS + PBS (1.67 ± 1.03 and 1.33 ± 0.82 vs. 3.00 ± 0.00 and 2.67 ± 0.82), but not PBS + iNSC pigs (2.67 ± 1.37 and 2.33 ± 1.51). HPS indicates hours post-stroke. * indicates a significant (*P* < .05) difference from the pre-stroke time point. # indicates a significant (*P* < .05) difference from the 0-4 HPS time point. $ indicates a significant (*P* < .05) difference from the 5 DPS and 1 DPT time points. † indicates a significant (*P* < .05) difference from the 2 and 3 DPT time points.

Clinically relevant modified Rankin Scale (mRS) assessments revealed severe functional deficits in post-stroke pigs as they were unable to independently ambulate or eat/drink due to hemiparesis and facial paralysis, thus requiring constant care (Fig. 6A top image; Fig. 6B mRS 5; Supplementary Table S1). At 2 days post-transplantation, Tan IIA-NP + iNSC treated pigs demonstrated functional recovery as they were capable of independent ambulation and eating/drinking with assistance (Fig. 6A middle image; Fig. 6B mRS 3; Supplementary Table S1). By 1 week post-transplantation, Tan IIA-NP + iNSC pigs demonstrated decreased mRS scores relative to 5 days post-stroke and 1 day post-transplantation, and again at 4 and 12 weeks post-transplantation relative to 2 and 3 days post-transplantation (Fig. 6A bottom image, Fig. 6B mRS score ≤ 2; Fig. 6C). Comparatively, PBS + iNSC and PBS + PBS treated pigs never demonstrated this level of neurological recovery (Fig. 6C). Treatment group comparisons at 1 week post-transplantation revealed significantly reduced mRS scores in Tan IIA-NP + iNSC treated pigs compared to PBS + PBS pigs (1.67 ± 1.03 vs. 3.00 ± 0.00), but not PBS + iNSC pigs (2.67 ± 1.37). These differences were preserved at 4 and 12 weeks post-transplantation as Tan IIA-NP + iNSC pigs again demonstrated a significant decrease in neurological deficits compared to PBS + PBS pigs (1.34 ± 0.81 vs. 2.67 ± 0.81), but PBS + iNSC pigs (2.34 ± 1.51), thus suggesting this novel combination therapy enhances functional stroke recovery.

## Discussion

With the ability to replace damaged neurons and glia as well as produce neuroprotective and regenerative growth factors, iNSCs possess significant therapeutic potential for ischemic stroke patients. However, the full therapeutic capacity of iNSCs has yet to be fully realized due to the relatively low level of iNSC engraftment in the cytotoxic post-stroke environment. This pivotal study presents the first experimental evidence that treatment with antioxidative and anti-inflammatory Tan IIA-NPs prior to iNSC transplantation improves iNSC survivability and engraftment as well as enhances cellular, tissue, and functional recovery in a translational pig ischemic stroke model. In this double-blinded study, pretreatment with Tan IIA-NPs increased iNSC neuronal differentiation and suppressed reactive astrocyte differentiation in the stroke tissue microenvironment. The novel combination therapy of Tan IIA-NPs and iNSCs attenuated tissue degradation and stimulated neural recovery processes as seen through increased endogenous neuronal cell survival, reduced immune cell activation, and gliosis as well as enhanced neurogenesis and neuroblast migration. These cellular changes resulted in reductions in lesion volumes, midline shift, white matter damage, cytotoxic edema, and hemorrhage all of which are critical prognostic indicators of functional recovery in patients.^34^ Ultimately, mRS neurological assessments showed that the Tan IIA-NP and iNSC combination therapy led to faster and improved neurological recovery in our translational pig ischemic stroke model. The findings of this study indicate that pretreatment with Tan IIA-NPs will lead to enhanced iNSC engraftment and the combination therapy will result in significant cellular, tissue, and functional recovery, thus making it a potentially transformative treatment for ischemic stroke patients.

Our research group recently demonstrated in a pilot study that Tan IIA-NPs may possess anti-inflammatory and antioxidative effects potentially resulting in decreased mean differences in cellular damage and improved mean differences in recovery in our pig ischemic stroke model.^25^ In the present and our previous study, Tan IIA was encapsulated into FDA approved PLGA-PEG NPs to protect Tan IIA from rapid degradation and enhance Tan IIA concentration in target tissues.^35^ Furthermore, the physiochemical and biological properties of NPs permit increased cellular uptake when compared to freeform molecules.^36^ These properties resulted in controlled extended release of Tan IIA that led to prolonged anti-inflammatory and antioxidative effects while reducing cytotoxicity. Intrathecal injection of Tan IIA-NPs into the subarachnoid space, rather than intravenous delivery, eliminated filtering by peripheral organs and allowed for the administration of lower Tan IIA doses (10-30 mg/kg intraperitoneal in rodents vs. 0.133 mg/kg intrathecal in pigs) and decreased dosage frequency.^24,25,37^ Intrathecal injection also bypassed the blood brain barrier that, despite stroke induced barrier disruptions, would significantly limit reliable delivery of therapeutics to ischemic tissues. NP delivery and intrathecal administration of Tan IIA was well tolerated by pigs and represents a novel approach in enhancing iNSC survivability post-transplantation.

Previous studies have shown that neural stem cells (NSCs) have a relatively low level of engraftment and long-term survival in rodent and pig ischemic stroke models. Darsalia et al showed in a rat MCAO model that 27-58% of transplanted human NSCs and derived differentiated cell types survived 6 weeks post-transplantation.^38^ Similarly, Mine et al showed that an average of 28% of NSCs survived at 4 weeks post-transplantation in a rat MCAO model.^39^ In our previous study evaluating the therapeutic effect of iNSCs in the pig MCAO model, cells were significantly more focal and localized to the injection site at acute time points and relatively low levels of iNSCs were observed 12 weeks post-transplantation.^5^ In this study, we demonstrated Tan IIA-NPs led to a 56% increase in iNSC and iNSC derived cell numbers 12 weeks post-transplantation. These results are likely a reflection of the antioxidative and anti-inflammatory neuroprotective effects of Tan IIA. In recent rodent stroke studies, Tan IIA increased antioxidant enzyme levels and reduced the generation of oxidative products resulting in decreased cell apoptosis.^18,20^ Tan IIA also caused increased levels of IL-4 and IL-13 and activation of the PI3K/Akt/mTOR survival signaling pathway.^21,22,40,41^ In this study, we demonstrated increased survivability of transplanted iNSCs and the most significant increases in endogenous neuron survival and decreases in Iba1 + immune cell activity in the brain tissues of Tan IIA-NP + iNSC treated pigs. The data from this study and previous studies support that Tan IIA-NP treatment led to improved survival and engraftment of iNSCs likely by creating a less cytotoxic stroke environment. We also believe Tan IIA-NPs may have improved iNSC migration and proliferation processes due the large distribution of HNA + cells at 12 weeks post-transplantation. These processes have been similarly observed in rodent stroke models.^6,8^

It has been previously theorized that improved iNSC survival would not only lead to enhanced cellular replacement of neurons and glia, but also increased neuroprotective and regenerative signaling. Prior studies have shown that NSC treatment leads to increased levels of BDNF, GDNF, NTF3, VEGF, and other neurotrophic factors which have demonstrated neuroprotective and regenerative effects in both rodent and pig stroke models.^5,42^ The data in this study further support the theory that increased NSC survival enhances neuroprotective and regenerative signaling as iNSC treatment without the Tan IIA-NP pretreatment resulted in a statistically significant increase in endogenous neuron survival and a decrease in immune cell and reactive astrocyte activation relative to PBS control pigs. However, administration of Tan IIA-NPs prior to iNSC transplantation resulted in a 50% and 127% increase in neuroprotection as measured by neuron survival and decreased Iba1 + immune cells, respectively, relative to iNSC only treatment. Similarly, iNSC treatment led to a significant increase in neurogenic activity at the vSVZ and LB relative to PBS control pigs. Again, Tan IIA-NP administration before iNSC transplantation resulted in a 30% and 32% increase in DCX + neuroblasts at the vSVZ and LB, respectively, as compared to iNSC only treated animals. Earlier rodent and pig reports have documented the neuroprotective, anti-inflammatory, and neurogenic effects of NSC treatment, yet this is the first report demonstrating the compounding effect of utilizing a Tan IIA-NP pretreatment.^43^

Lesion volume and midline shift are valuable predictors of patient prognosis due to their strong correlation with neurological deficits.^34,44^ Although previous studies have shown mixed results with respect to the ability of NSCs to reduce lesion volumes, it has been shown that there is a strong correlation between increased NSC engraftment and decreased lesion volume.^45^ Here, the Tan IIA-NP + iNSC treatment that increased NSC survivability and engraftment also significantly decreased lesion volumes and midline shift 12 weeks post-transplantation, thus indicating a significant reduction in tissue damage and atrophy. Conversely, in our previous study in which post-stroke pigs were only treated with iNSCs, we did not observe a significant decrease in lesion volume.^5^ The results from these studies suggest Tan IIA-NP + iNSC treatment is more effective at mitigating tissue damage and atrophy than iNSCs alone. The correlation between NSC treatment and improved neurological outcomes has been well documented in both rodent models^6, 7, 9, 10^ and clinical trials of ischemic stroke.^46,47^ Tan IIA-NP + iNSC-treated pigs demonstrated neurological recovery by 2 days post-transplantation, likely due to iNSC secreted trophic factors. By 4 and 12 weeks post-transplantation, Tan IIA-NP + iNSC pigs demonstrated a further decrease in mRS scores relative to 2 and 3 days post-transplantation, whereas iNSC only and PBS pigs never demonstrated this level of recovery. Interestingly, patients who received Tan IIA injections demonstrated similar functional recovery as indicated by mRS scores ≤ 1 12 weeks post-stroke.^48^ Collectively, these findings suggest increased iNSC survival and engraftment, afforded by antioxidative and anti-inflammatory Tan IIA-NPs, led to improved cellular and tissue recovery and ultimately enhanced neurological recovery.

## Conclusion

For the first time, this study demonstrated pretreatment with antioxidative and anti-inflammatory Tan IIA-NPs created a less cytotoxic post-stroke microenvironment that enhanced the multimodal cell replacement, neuroprotective, and regenerative effects of iNSCs on cerebral tissue and functional recovery in a clinically relevant pig ischemic stroke model. Tan IIA-NP + iNSC treatment resulted in comprehensive therapeutic efficacy ranging from increased neuronal survival and neurogenesis and decreased immune cell activation to reduced lesion volumes, midline shift, and neurological deficits. These results must be interpreted while considering the limitations of this study including the need for additional MRI analysis immediately prior to iNSC transplantation as transplantation depths may be affected by subacute lesion and edema evolution. Nevertheless, these findings collectively support the continued investigation of this novel combination therapy for potential translation to clinical trials.

## Supplementary Material

szac062_suppl_Supplementary_Material

## Data Availability

The datasets generated during the current study are available from the corresponding author upon request.
