# Supplementary material for: Tanshinone IIA-Loaded Nanoparticle and Neural Stem Cell Therapy Enhances Recovery in a Pig Ischemic Stroke Model
Source: Stem Cells Transl Med. 2022 Sep 19;11(10):1061–71. doi: 10.1093/stcltm/szac062 (PMC9585947; doi:10.1093/stcltm/szac062)
Supplement: szac062_suppl_Supplementary_Material [file szac062_suppl_supplementary_material.docx]

**Supplemental materials**

**Detailed methods**

**Animals and housing**

This study was performed in accordance with the National Institutes of Health (NIH) Guide for the Care and Use of Laboratory Animals guidelines and approved by the University of Georgia Institutional Animal Care and Use Committee (IACUC; Protocol Number: 2017-07-019Y1A0). Twenty-two, nine-month-old, sexually mature, castrated male, Yucatan miniature swine 31-41 kg were purchased from Lonestar™ Laboratory Swine. These animals were selected in accordance with the Stem Cell Emerging Paradigm in Stroke (STEPS) and Stroke Therapy Academic Industry Roundtable (STAIR) consortium recommendations of performing initial therapeutic evaluations in “healthy males” of a “higher-order gyrencephalic species” that is “more reflective of human pathology”.^1-3^ Pigs were group housed prior to stroke induction in a Public Health Service (PHS) and American Association for Accreditation of Laboratory Animal Care (AAALAC) approved facility at 27°C with a 12 hour light/dark cycle. Pigs were given free access to water and fed standard grower diets with daily enrichment provisions.

**Synthesis of Poly(lactide-*co*-glycolide)-b-poly(ethylene glycol) (PLGA-b-PEG-OH) polymer**

Poly(D,L-lactide-*co*-glycolide)-COOH (PLGA-COOH, 1.0 g, 0.170 mmol, Lactel), poly(ethylene glycol) (OH-PEG-OH, 2.29 g, 0.684 mmol, Sigma Aldrich), and 4-(Dimethylamino)pyridine (DMAP, 0.023 g, 0.187 mmol, Alfa Aesar) were dissolved in 30 mL of anhydrous dichloromethane (CH_2_Cl_2_). 10 mL CH_2_Cl_2_ solution of N,N’-Dicyclohexylcarbodiimide (DCC, 0.141 g, 0.684 mmol, Sigma Aldrich) was added dropwise to the reaction mixture at 0°C and continuously stirred with a magnetic stir bar. The reaction mixture was warmed to 25°C and stirred overnight. Insoluble dicyclohexylurea (C_13_H_24_N_2_O) was filtered out. The raw product was precipitated out by adding 50 mL of 1:1 diethyl ether ((C_2_H_5_)_2_O) and methanol (CH_3_OH) to the reaction mixture. The mixture was centrifuged for 15 minutes at 4°C. The purification step was repeated 4-5 times, followed by ^1^H NMR analysis (Varian Mercury Plus 400).

**Synthesis and characterization of Tanshinone-IIA nanoparticles (Tan IIA-NPs)**

Poly(lactide-*co*-glycolide (PLGA) nanoparticles (NPs) were synthesized through a nanoprecipitation method.^4, 5^ Briefly, polymer (PLGA-b-PEG-OH) was dissolved in dimethylformamide (DMF) at a concentration of 50 mg/mL. 100 μL of the polymer solution was mixed with Tan IIA (150 μL, 0.15 mg) at 30% of drug feeding with respect to polymer concentration and diluted with DMF to a final polymer concentration of 5 mg/mL. The mixture was added dropwise to sterilized nanopure water with constant stirring and the resulting solution was agitated in a fume hood for 2 hours. Tan IIA-NPs were collected on an amicon ultracentrifugation unit (100 kDa cut-off) and were washed 3-4 times with water. Finally, the NPs were resuspended in sterilized nanopure water.

A drop of diluted NP solution in water was deposited onto a transmission electron microscopy (TEM) grid, followed by staining with 2% uranyl acetate. A TEM image was taken on an FEI Tecnai 20 transmission electron microscope operating at an accelerating voltage of 200 kV. Hydrodynamic size and surface charge of NPs were analyzed on a Malvern Zetasizer Nano ZS system.

To evaluate drug loading capacity, a 50 μL aqueous solution of NPs was diluted to 900 µL. 100 μL 0.1 mM sodium hydroxide (NaOH) was added to the solution and left at 23°C overnight. Afterwards, the solution was sonicated for 30 minutes and centrifugated at 5000 rpm for 10 minutes. 100 μL supernatant was transferred into a 96-well ultraviolent (UV) transparent plate and its absorbance at 258 nm was measured.

**Human induced pluripotent stem cell derived neural stem cell (iNSC) culture**

SOX/Nestin+ iNSCs (**Figure S1)**, which differentiate into βIII*-*tubulin*+* neurons, GFP+ astrocytes, and Olig2+ oligodendrocytes in vitro, were maintained on Matrigel-coated (Corning) tissue culture plates in neural stem cell media composed of Neurobasal medium (Gibco), 2% B-27 Supplement (Gibco), 1% non-essential amino acids (Gibco), 2 mM L-glutamine (Gibco), 1% penicillin/streptomycin (Gibco), 20 ng/mL bFGF (R&D Systems). A complete media change was performed every other day. When iNSCs reached 90% confluence, cells were enzymatically passaged using Accutase (Gibco). To track transplanted iNSCs, 3 days prior to transplantation, 1.4x10^7^ iNSCs were labeled with 1,1′-dioctadecyl-3,3,3′,3′-tetramethylindotricarbocyanine iodide (DiR) at a concentration of 2 µg/mL diluted in phosphate buffered saline (PBS). After incubation at 37°C for 5 minutes, iNSCs were washed twice with PBS, then washed once with media via centrifugation at 250 x g. Cells were re-plated on Matrigel-coated (Corning) tissue culture treated plates at 7.0x10^6^cells per 150 mm plate and maintained in neural stem cell media as described above until transplantation.

**Preoperative, perioperative, and postoperative procedures**

One day prior to stroke induction, pigs were administered ceftiofur crystalline free acid [5 mg/kg, intramuscular (IM)] and fentanyl [100 mg/kg/hr, transdermal (TD)] for infection prevention and pain management, respectively. Preoperative analgesia and sedation were achieved using xylazine (2 mg/kg, IM) and midazolam (0.2 mg/kg, IM). To facilitate endotracheal intubation, propofol [10 mg/mL, intravenous (IV)] and prophylactic lidocaine (2%, topically to the laryngeal folds) were administered to effect. Lactated ringer’s solution was administered (5 mL/kg/hour, IV) to maintain hydration.

Anesthesia was maintained with isoflurane (1.0-2.0%) in oxygen and air with vitals including temperature, respiration, heart rate, and blood pressure continuously monitored and maintained within normal parameters. Artificial ventilation was maintained at 8-12 breaths per minute with a tidal volume of 5-10 mL/kg. Heart rate was monitored by Doppler probe placement on the femoral artery while blood pressure was monitored by a sphygmomanometer. Rectal temperature was recorded every 15 minutes using a digital thermometer.

Following surgical procedures, anesthesia was discontinued and pigs were returned to their pens upon extubation and monitored every 15 minutes until vitals (temperature, heart rate, respiration rate) returned to normal, every 4 hours for 24 hours, and twice a day for the remainder of the study. Flunixin meglumine (2.2 mg/kg, IM) was administered for postoperative pain and fever management every 12 hours for the first 24 hours, and every 24 hours for 3 days post-stroke. **Middle cerebral artery occlusion (MCAO) surgical procedures**

As previously described by Platt et al. a right rostrotentorial craniectomy approach was utilized to access the middle cerebral artery (MCA).^6^ Briefly, a frontotemporal curvilinear skin incision exposed the fascia and muscle. The zygomatic arch was partially resected, thus exposing the ventral aspect of the calvaria. A surgical defect was generated in the calvaria and the visible dura mater was reflected. The arachnoid was opened and the distal portion of the MCA was permanently cauterized utilizing bipolar forceps to induce ischemic infarction spanning the most caudal aspect of the frontal lobe, significant areas of the temporal lobe, and portions of the parietal and occipital lobes. The exposed brain was then covered with a sterile biograft made of porcine small intestine submucosa (MatriStem, ACell) and the temporalis muscle and epidermis were routinely sutured.

**Tan IIA-NP administration**

One hour post-stroke, a 20 gauge 3.5” spinal needle was inserted at the midline of the dorsal neck at an anatomical intersection created by a vertical line of the rostral aspect of the wings of the first vertebral body and a horizontal line connecting the dorsal arch of C2 with the occipital protuberance. Once the needle was through the cutaneous tissues, the stylet was removed and advanced until cerebrospinal fluid (CSF) appeared in the needle hub confirming entry into the cerebellomedullary cistern. A small volume (3-5 mL) of CSF was removed while the spinal needle was in place and the volume removed was replaced with an identical volume of PBS or Tan IIA-NPs. Each pig received a dose of 133 μg/kg Tan IIA-NPs per previous findings.^4^

**iNSC transplantation**

Five days post-stroke, all pigs received a transcranial transplantation of either iNSC treatment or PBS. Pigs were anesthetized according to the aforementioned preoperative and anesthesia protocols. Transplantation surgeries were performed as previously described by Baker et al. utilizing a stereotaxic frame (David Kopf Instruments) with specific pig modifications.^7^ A mounted quintessential stereotaxic injector (Stoelting) was utilized to inject 1.2x10^7^ DiR-labeled iNSCs at a rate of 2 µL/minute to prevent backflow. Immediately prior to injection, approximately 80 µL of iNSCs suspended in PBS or PBS-only control were sterilely loaded into a glass micropipette syringe with a 26 gauge needle (Hamilton) and attached to the stereotaxic apparatus.

Transplantation depth was determined via coronal T2Weighted MRI sequences. Specifically, the most caudal T2Weighted image in which the craniectomy site was visualized was utilized to measure the distance from the most dorsal aspect of the craniectomy site within the perilesional region to the center of the white matter utilizing Osirix imaging software (**Fig S4**). Transplantation depths were individually calculated with an average depth of 2.02±0.21 cm which was equally subdivided into four separate injection depths. This permitted the injection of 4 boli, approximately 20 µL each, into both white and gray matter compartments spanning inferior to superior relative to the cortex. After transplantation was complete, the needle was retracted at a rate of 1 mm/minute to prevent backflow and the epidermis was sutured. Anesthesia was discontinued and the pigs were returned to their pens upon extubation. Again, pigs were monitored every 15 minutes until vitals (temperature, heart rate, respiration rate) returned to normal, every 4 hours for 24 hours, and twice a day for the remainder of the study.

**Magnetic resonance imaging (MRI) acquisition and analysis**

MRI was performed 24 hours post-stroke and 12 weeks post-transplantation using a General Electric 3.0 Tesla MRI system. Pigs were sedated and maintained under anesthesia as previously described for pre-, peri-, and postoperative procedures. MRI of the cranium was performed using an 8 channel torso coil with the pig positioned in supine recumbency. Multiplanar MRI sequences were acquired including T2 Weighted (T2W), T2 Fluid Attenuated Inversion Recovery (T2FLAIR), T2 Star (T2*), Diffusion Weighted Imaging (DWI), and Diffusion Tensor Imaging (DTI). T2W, T2FLAIR, T2*, DWI and apparent diffusion coefficient (ADC) maps were analyzed using OsiriX software, while DTI generated fractional anisotropy (FA) maps were analyzed using ImageJ software.

T2FLAIR, DWI, and ADC maps were collectively utilized to confirm the presence of ischemic stroke 1-day post-stroke. Specifically, cytotoxic edema consistent with ischemic stroke was qualitatively confirmed by comparing corresponding hyperintense regions of interest (ROIs) in T2FLAIR and DWI sequences with hypointense ROIs in DWI generated ADC maps.

Lesion volumes were semi-automatically calculated utilizing T2W sequences. Specifically, ischemic lesions defined by hyperintense ROIs were manually outlined at each transverse slice by trained blinded analysts utilizing OsiriX software. Once all lesion areas were defined, computer generated lesion volumes were automatically calculated and reported in cm^3^. Midline shift was also calculated utilizing T2W sequences for each transverse slice by measuring the distance from the natural midline along the anterior and posterior attachments of the falx cerebri to the septum pellucidum.^8^ The distance between the exact midpoint of the length of the septum pellucidum and the ideal midline was measured and reported in mm.

FA maps were utilized to assess changes in white matter integrity. Specifically, the ipsilateral and contralateral internal capsules were identified at each transverse slice by trained blinded analysts. Then, ImageJ generated mean FA values were recorded for the ipsilateral and contralateral internal capsules. Changes in the mean FA value of the ipsilateral internal capsule are expressed as a percent change relative to the contralateral hemisphere.

ADC maps were utilized to assess changes in diffusivity within ischemic lesions. Specifically, ischemic lesions defined by hypointense ROIs in the ipsilateral hemisphere were manually identified at each transverse slice by trained blinded analysts. Then, identical OsiriX generated ROIs in the contralateral hemisphere were identified at each transverse slice. OsiriX generated mean ADC values for both the ipsilateral and contralateral hemispheres and changes in the mean ADC value of the ipsilateral hemisphere are expressed as a percent change relative to the contralateral hemisphere.

Intracerebral hemorrhage (ICH) volume was calculated utilizing T2* sequences by manually identifying hypointense ROIs in the ipsilateral hemisphere at each transverse slice. OsiriX generated ICH volumes are reported as cm^3^. ICH incidence was also confirmed by a board certified veterinary pathologist via histological analyses. As ICH has not been previously observed in all pigs in previous studies^6, 9^, ICH volumes herein likely correspond to the level of blood brain barrier disruption, inflammatory response, and lesion volumes as commonly seen in patients.

**Neurological assessment**

Pigs were assessed by a blinded, trained rater and assigned a modified Rankin Scale (mRS) score pre-stroke; 0-4, 4-8, 8-12, 12-16, 16-20, 20-24 hours post-stroke; 2, 3, 4, and 5 days post-stroke; 1, 2, and 3 days post-transplantation; and 1, 4, and 12 weeks post-transplantation.^8^ Possible scores ranged from 0 (no residual stroke symptoms) to 6 (death) and were evaluated across the entire score range as a noncontinuous variable. When assigning mRS scores, pigs were assigned to an mRS category according to the classification of a pig’s worst stroke symptom. For example, if a pig required assistance to stand/walk (mRS 4) and assistance to eat/drink (mRS 3), the pig would be assigned an mRS score of 4 as one of the pig’s disabilities still categorizes it as having a moderate to severe disability. This clinical scale captured functional recovery in post-stroke pigs and is further described in **Table S1**.

**Brain tissue collection and processing**

Following acquisition of 12 week post-transplantation MRI, pigs were euthanized via euthanasia solution (1mL/10 lbs, IV) and brains were removed. For iNSC transplantation identification, whole brain and coronal sections were imaged using an IVIS Lumina II System 136 (PerkinElmer, Waltham, MA). DiR fluorescence was visualized using a 745 nm excitation wavelength and an Indocyanine Green (ICG) emission filter. Three consecutive coronal sections of high DiR fluorescence were collected, fixed in 4% paraformaldehyde (PFA, Electron Microscopy Sciences) for 24 hours, dehydrated in 30% sucrose for 2 weeks, embedded in Tissue-Tek OCT compound (Sakura), and stored at -80°C. Remaining brain sections were preserved for immunohistochemistry analysis and immersed in 10% buffered formalin.

**Immunofluorescent staining**

OCT embedded, fixed tissues were cryosectioned into 10 µm sections and mounted onto 3x5 inch gelatin subbed glass slides. Slide mounted tissue sections were stored at -20°C. Prior to staining, slides were thawed at room temperature for 30 minutes. Slides were washed with PBS to remove excess OCT and rehydrate tissues and quenched with 0.25 M glycine solution for 1 hour. Tissues were then washed with PBS containing 0.5% Triton X-100 (PBS-x) 3 times for 5 minutes each. Tissues were blocked in 6% normal donkey serum in PBS-x for 1 hour. Primary and secondary antibodies were diluted in 6% normal donkey serum in PBS-x. Tissues were washed with PBS-x after blocking; before primary antibody anti-HNA (mouse, 1:1000, abcam ab191181); and before one of the following anti-NeuN (guinea pig, 1:750, Sigma-Aldrich ABN90P), anti-GFAP (chicken, 1:500, abcam ab4674), or anti-Olig2 (rabbit, 1:250, GeneTex GTX132732). Slides were incubated overnight at 4°C. Tissues were washed with PBS-x and blocked again for 1 hour. Secondary antibodies, anti-mouse Alexa Fluor 488 (1:1000, A11029), anti-guinea pig Alexa Fluor 594 (1:1000, A11076), anti-chicken Alexa Fluor 594 (1:500, A11042), and anti-rabbit Alexa Fluor 594 (1:500, A11037) were incubated in the dark for 1 hour at room temperature. Finally, tissues were thoroughly washed with PBS before being mounted in Prolong Gold with DAPI (Invitrogen) and allowed to cure overnight before microscopic inspection.

Images were collected on a Cytation 5 reader (Biotek Instruments, Inc.). Representative z-stack images were taken at 4x and 10x, while whole-hemisphere montage images were captured at 4x and 10x, utilizing Gen5 Software (Biotek Instruments, Inc.) and digitally stitched together to create a single image. Cell quantification was performed on whole hemisphere images using ImageJ software. Staining, imaging, and analysis was performed in triplicate for each neural marker (i.e., NeuN, GFAP) for each pig.

**Immunohistochemistry**

Heat induced antigen retrieval was performed for all antibodies using citrate buffer at pH6 (DAKO). Detection was performed utilizing biotinylated antibodies and a streptavidin label (4plus Streptavidin HRP, Biocare). A HRP label and DAB chromogen (DAKO) were used with all sections counterstained with hematoxylin. For NeuN, Iba1, and GFAP images were taken along the lesion border (LB) of the whole ipsilateral hemisphere. For DCX, three separate anatomical regions were analyzed at the level of the caudate nucleus: 1. the neuroblasts positioned directly adjacent to the ependymal cells lining the lateral ventricle (ventricular subventricular zone; vSVZ), 2. the neuroblasts organized laterally into chains adjacent to, but distinct from the vSVZ (abventricular SVZ; aSVZ), and 3. the neuroblasts that migrated laterally to the LB. NeuN and DCX positive cells were manually quantified using ImageJ 2.0 software and expressed as cells/mm^2^. For semi-quantitative analysis of Iba1 and GFAP, the total area of immunoreactivity corresponding to increased optical density was determined by ImageJ and expressed as percent positive area.

**Statistical analysis**

All quantitative data was analyzed with SAS version 9.3 (Cary, NC). Statistical significances between groups were determined by one-way ANOVA and post-hoc Tukey-Kramer Pair-Wise comparisons with p-values<0.05 considered significantly different. A linear mixed model was used to determine statistical significances between neurological assessment time points. The full model included fixed effects for treatment and time, a treatment by time interaction, and a random intercept for each pig which was included to account for within pig correlation. Simple treatment effects at each time were performed and multiple comparisons were adjusted for using Tukey’s test.

**Supplemental figures**

##
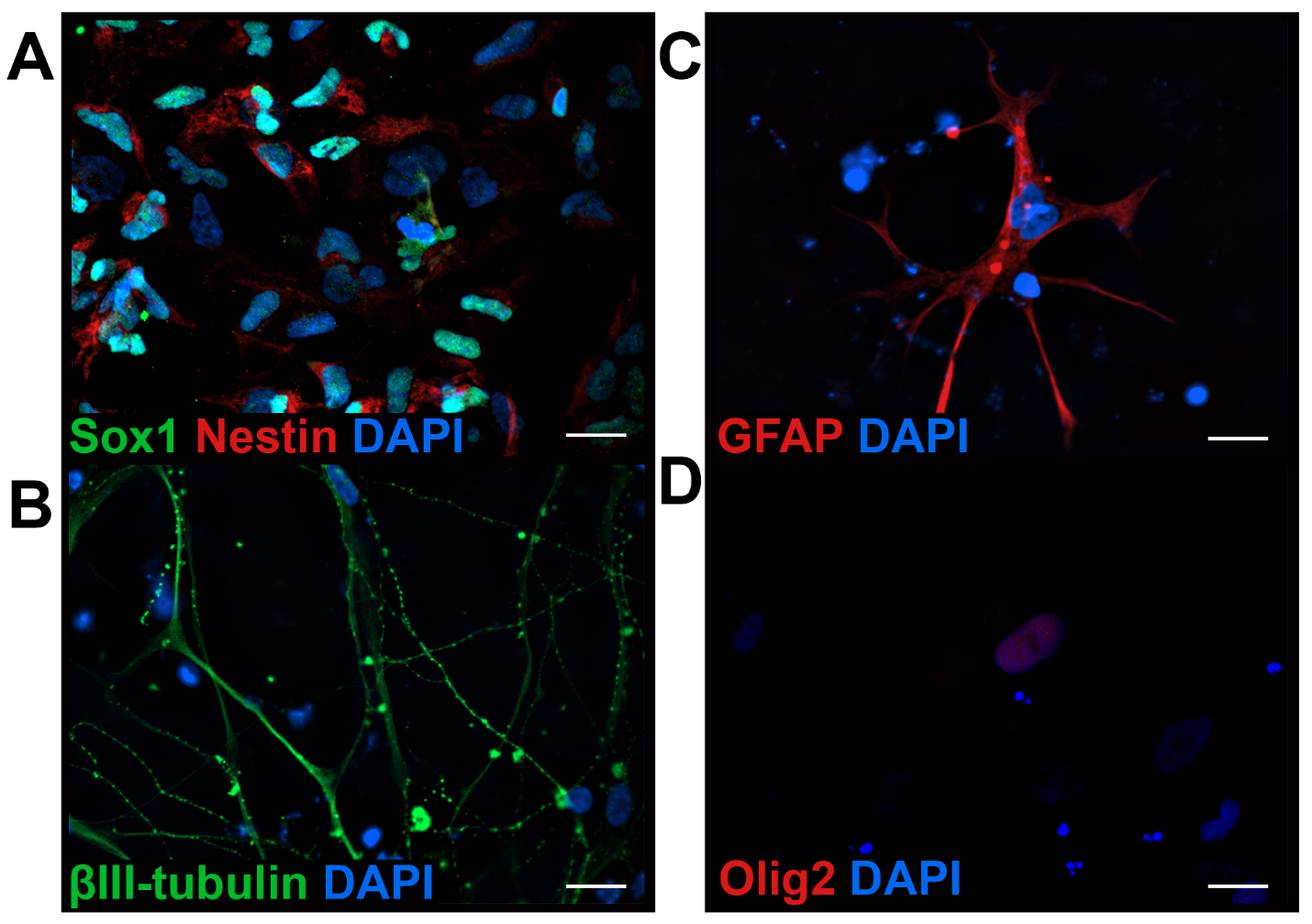


**Figure S1. iNSCs differentiate into neurons, astrocytes, and oligodendrocytes *in vitro*.** SOX1+/Nestin+ iNSCs (**A**) are capable of spontaneous differentiation into βIII*-*tubulin+ neurons (**B**), GFP+ astrocytes (**C**), and Olig2+ oligodendrocytes in vitro (**D**). Scale bars 20 µm.


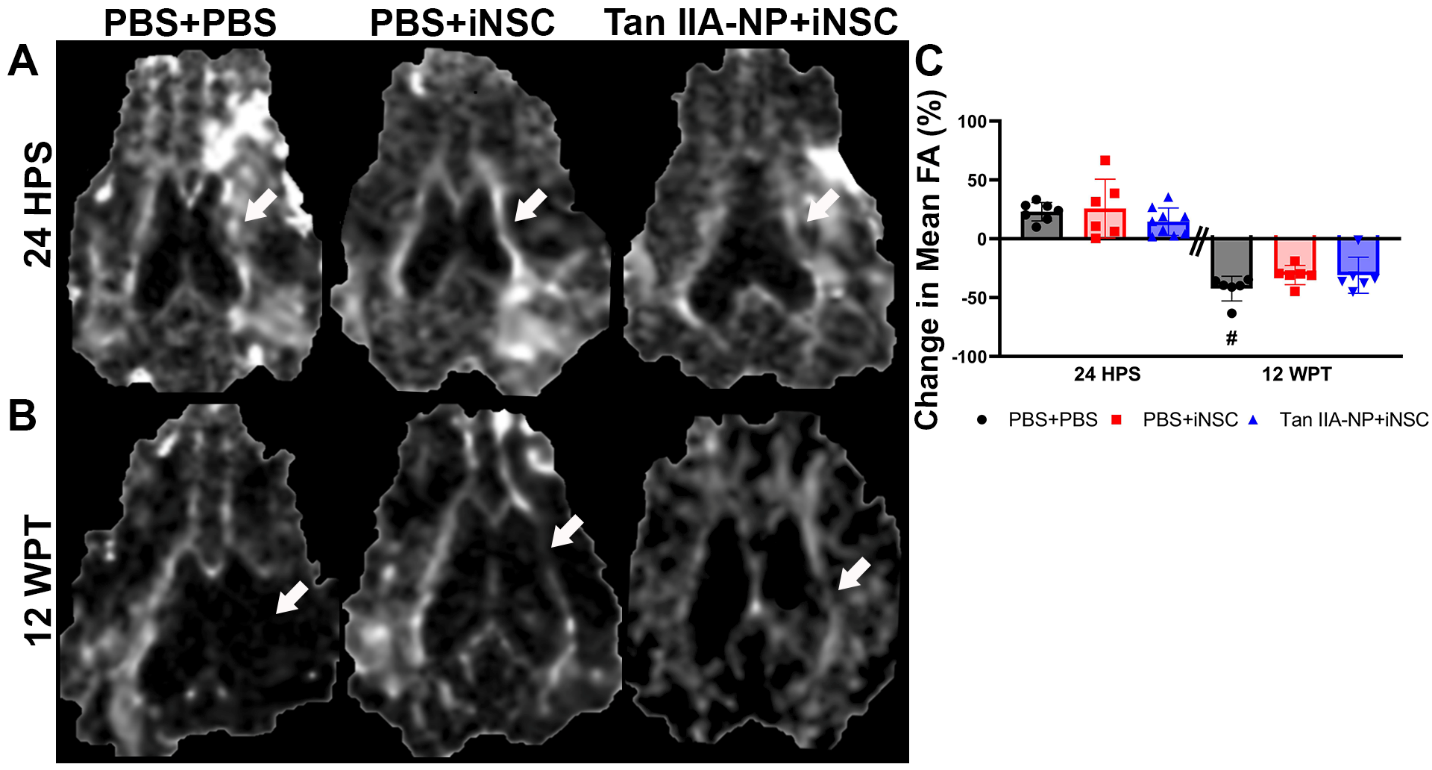


**Figure S2. iNSCs prevented chronic white matter degradation.** Fractional anisotropy (FA) maps depicted territorial changes in the ipsilateral internal capsule (white arrows) 24 hours post-stroke and 12 weeks post-transplantation (24 HPS, 12 WPT, respectively; **A**, **B**). Tan IIA-NP+iNSC and PBS+iNSC treated pigs exhibited a lower percent decrease in mean FA values compared to PBS+PBS pigs at 12 WPT (-30.90±13.96 vs. -30.70±7.47 vs. -42.25±9.65%, respectively; **C**), although not statistically significant (p=0.1). Although PBS+iNSC treated pigs exhibited the greatest percent change in FA values at 24 hours post-stroke, they show comparable FA values to Tan IIA-NP+iNSC treated pigs 12 weeks post-transplantation. This suggests iNSC transplantation may help prevent white matter degradation following ischemic injury. This hypothesis is reinforced as PBS+PBS control pigs demonstrated a significant (p<0.05) difference in percent change between time points (-42.25±9.65 vs. 23.01±7.25%, respectively; **C**), whereas Tan IIA-NP+iNSC and PBS+iNSC treated pigs did not. Data are expressed as mean±SD # indicates a significant (p<0.05) difference between time points. At 24 HPS PBS+PBS data n=7, PBS+iNSC data n=6, and Tan IIA-NP+iNSC data n=9. At 12 WPT PBS+PBS data n=6, PBS+iNSC data n=6, and Tan IIA-NP+iNSC data n=6.


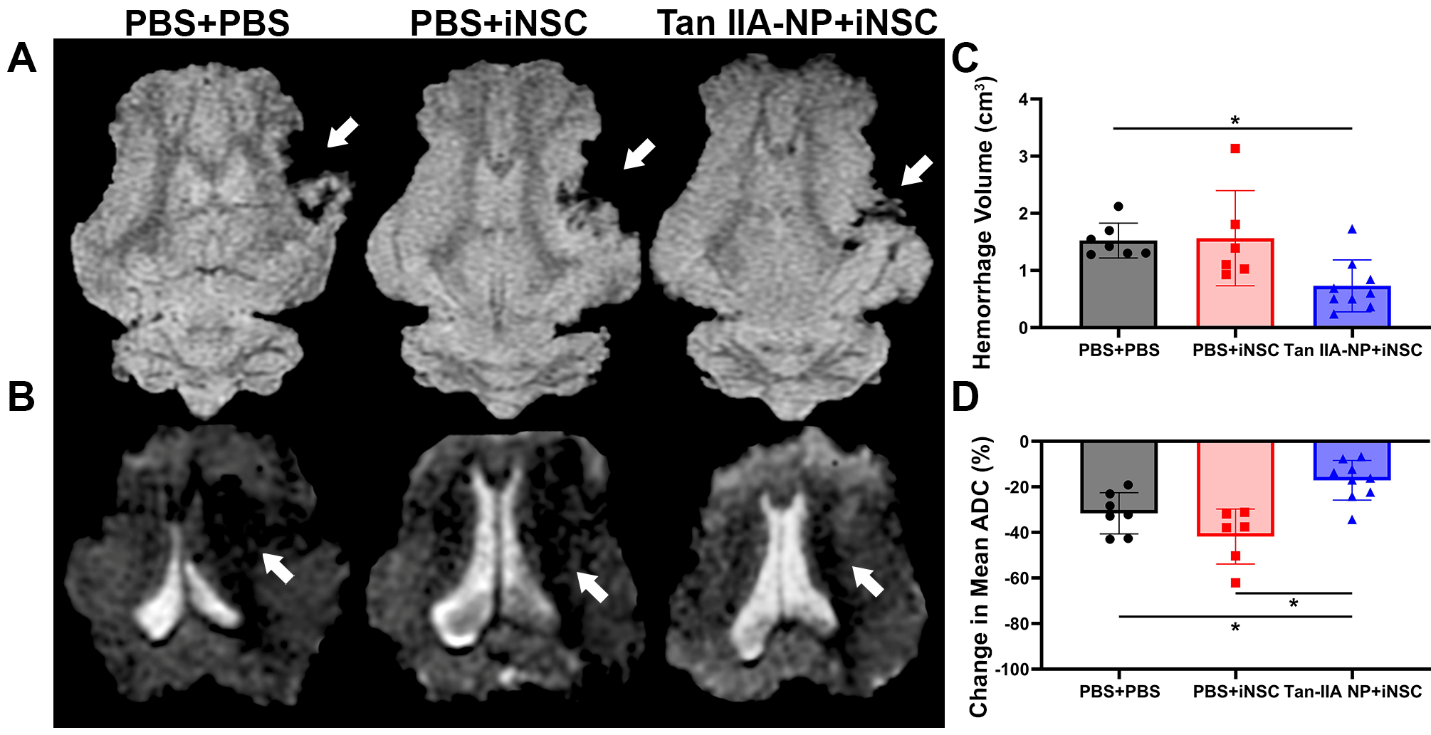


**Figure S3. Tan IIA-NP treatment reduced intracerebral hemorrhage and preserved diffusivity.** T2* sequences revealed PBS+PBS and PBS+iNSC pigs exhibited greater intracerebral hemorrhage as indicated by hypointense regions of interest (white arrows; **A**) relative to Tan IIA-NP+iNSC pigs 24 hours post-stroke (1.52±0.28 vs. 1.90±1.48 vs. 0.73±0.43cm^3^, respectively; **C**), thus suggesting Tan IIA-NPs preserved the integrity of microvessels and associated capillary beds. Cerebral diffusivity was evaluated utilizing DWI sequences and derived apparent diffusion coefficient (ADC) maps. Signal void, consistent with restricted diffusion and indicative of cytotoxic edema was quantified (white arrows; **B**) 24 hours post-stroke and mean ADC values in the ipsilateral hemisphere were compared to the contralateral hemisphere with percent changes closer to zero being more similar to normal tissue. Tan IIA-NP+iNSC pigs exhibited a lower percent decrease in mean ADC (−17.11±8.20%; **B**, **D**) relative to PBS+PBS and PBS+iNSC pigs (−31.58±8.40 vs. −41.81±11.05%, respectively), thus indicating that Tan IIA-NPs preserved diffusivity and decreased cytotoxic edema as iNSCs were not transplanted until 5 days post-stroke. Data are expressed as mean±SD. * indicates a significant (p<0.05) difference between experimental groups. PBS+PBS data n=7, PBS+iNSC data n=6, and Tan IIA-NP+iNSC data n=9.

**
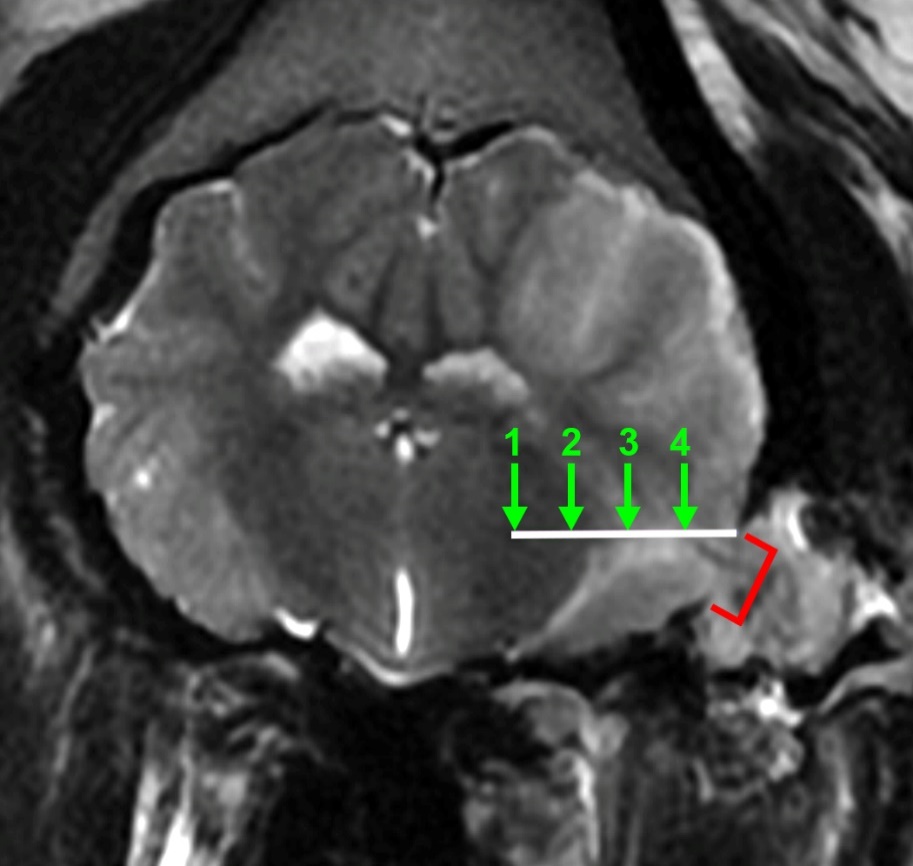
**

**Figure S4. Stereotaxic iNSC transplantation utilized clinically relevant MRI and Osirix imaging software technologies.** iNSC transplantation depth (white line) was calculated utilizing the most caudal T2W slice in which the craniectomy site (red bracket) was visualized. In this slice, the distance from the most dorsal aspect of the craniectomy site within the perilesional region to the center of the white matter was measured utilizing Osirix imaging software. This distance was then equally subdivided to permit the injection of 4 iNSC boli (green arrows) into white and gray matter compartments spanning inferior to superior relative to the cortex.

**Supplemental table**

|  | | **mRS Score** |
| --- | --- | --- |
| **Deceased** | Yes | **6** |
|  | No | **0-5** |
| **General symptoms** | No symptoms | **0** |
|  | No significant disability | **1** |
|  | Requiring consistent care and attention | **2-5** |
| **Food/Drink** | Eating without assistance | **0-2** |
|  | Drinking without assistance | **0-2** |
|  | Eating with assistance | **3** |
|  | Drinking with assistance | **3** |
|  | Not eating or drinking | **5** |
|  | Facial paralysis | **4** |
| **Motor function** | Walking without assistance | **0-3** |
|  | Standing without assistance | **0-3** |
|  | Walking with assistance | **4** |
|  | Standing with assistance | **4** |
|  | Circling to the right | **4** |
|  | Circling to the left | **4** |
|  | Unable to stand and move on own | **5** |
| **Bodily function** | Urination | **0-3** |
|  | Defecation | **0-3** |
| **Level of consciousness** | Awake | **0-4** |
|  | Asleep | **0-4** |
|  | Sedated | **5** |
|  | Requiring constant care and attention | **5** |

**Table S1. Clinical modified Rankin Scale (mRS) descriptions.**

**References**

1. Lapchak PA, Zhang JH, Noble-Haeusslein LJ. Rigor guidelines: Escalating stair and steps for effective translational research. *Translational stroke research*. 2013;4:279-285

2. Savitz SI, Chopp M, Deans R, Carmichael T, Phinney D, Wechsler L, et al. Stem cell therapy as an emerging paradigm for stroke (steps) ii. *Stroke*. 2011;42:825-829

3. Fisher M, Feuerstein G, Howells DW, Hurn PD, Kent TA, Savitz SI, et al. Update of the stroke therapy academic industry roundtable preclinical recommendations. *Stroke*. 2009;40:2244-2250

4. Waters ES, Kaiser EE, Yang X, Fagan MM, Scheulin KM, Jeon JH, et al. Intracisternal administration of tanshinone iia-loaded nanoparticles leads to reduced tissue injury and functional deficits in a porcine model of ischemic stroke. *IBRO Neurosci Rep*. 2021;10:18-30

5. Gao S, Zhang W, Wang R, Hopkins SP, Spagnoli JC, Racin M, et al. Nanoparticles encapsulating nitrosylated maytansine to enhance radiation therapy. *ACS Nano*. 2020;14:1468-1481

6. Platt SR, Holmes SP, Howerth EW, Duberstein KJ, Dove CR, Kinder HA, et al. Development and characterization of a yucatan miniature biomedical pig permanent middle cerebral artery occlusion stroke model. *Exp Transl Stroke Med*. 2014;6:5

7. Baker EW, Platt SR, Lau VW, Grace HE, Holmes SP, Wang L, et al. Induced pluripotent stem cell-derived neural stem cell therapy enhances recovery in an ischemic stroke pig model. *Sci Rep*. 2017;7:10075

8. Spellicy SE, Kaiser EE, Bowler MM, Jurgielewicz BJ, Webb RL, West FD, et al. Neural stem cell extracellular vesicles disrupt midline shift predictive outcomes in porcine ischemic stroke model. *Transl Stroke Res*. 2020;11:776-788

9. Webb RL, Kaiser EE, Jurgielewicz BJ, Spellicy S, Scoville SL, Thompson TA, et al. Human neural stem cell extracellular vesicles improve recovery in a porcine model of ischemic stroke. *Stroke*. 2018;49:1248-1256
